# Anterior segment pars plana vitrectomy combined with posterior capsulorhexis, phacoemulsification and trabeculectomy in treatment of medically uncontrolled acute primary angle-closure glaucoma: A retrospective study

**DOI:** 10.1097/MD.0000000000030946

**Published:** 2022-10-07

**Authors:** Jie Qu, Zhen Tian, Xin Li, Yong Zhang

**Affiliations:** a Department of Ophthalmology, Taihe Hospital, Hubei University of Medicine; b Jinzhou Medical University

**Keywords:** acute primary angle-closure glaucoma, anterior segment pars plana vitrectomy, Intraocular pressure, medically uncontrolled

## Abstract

To explore the clinical efficacy of quadruple surgery (anterior segment pars plana vitrectomy + phacoemulsification + posterior capsulorhexis + intraocular lens (IOL) implantation + trabeculectomy) and dual surgery (phacoemulsification + IOL implantation + trabeculectomy) to treat medically uncontrolled acute primary angle-closure glaucoma (APACG). The clinical data of 44 patients (45 eyes) with APACG treated in the Department of Ophthalmology of Taihe Hospital were retrospectively analyzed. They were divided into 2 groups based on quadruple surgery and dual surgery. There were 20 patients (20 eyes) underwent quadruple surgery in group A. And there were 24 patients (25 eyes) dual surgery in group B. The changes in intraocular pressure (IOP), visual improvement, and complications were observed between the 2 groups preoperatively and 1 month, 3 months, and 6 months postoperatively. Preoperative best corrected visual acuity (BCVA) was the influencing factor of postoperative BCVA at 1 month, 3 months and 6 months. Before surgery, the mean IOP of group A was significantly higher than that of group B (*P* < .001), and no significant difference was found in the BCVA, age, gender, eyes, axial length (AL), anterior chamber depth (ACD) (*P*_BCVA_ = 0.12, *P*_age_ = 0.76, *P*_eyes_ = 0.20, *P*_gender_ = 0.37, *P*_AL_ = 0.94, *P*_ACD_ = 0.08). On comparison at postoperative 1week, there was no significant difference in the IOP and BCVA between the 2 groups (*P*_IOP_ = 0.64, *P*_BCVA_ = 0.66). The mean IOP of group A was significantly lower than that of group B 1 month, 3 months, and 6 months postoperatively (*P*_1month_ = 0.002, *P*_3months_ < 0.001, *P*_6months_ < 0.001). The degree of visual acuity recovery was significantly higher in group A at 1 month, 3 months, and 6 months postoperatively (*P*_1month_ = 0.03, *P*_3months_ = 0.02, *P*_6months_ = 0.02). During treatment, the incidence of complications in group B was significantly higher than that in group A (*P* < .01). The clinical efficacy of anterior segment pars plana vitrectomy combined with posterior capsulorhexis, phacoemulsification, and trabeculectomy elicits clinical safety in treating medically uncontrolled APACG. It has remarkable effects and leads to a significant decrease in the occurrence of complications.

## 1. Introduction

Acute primary angle-closure glaucoma (APACG) is an ophthalmological disease caused by the sudden closure of the anterior chamber angle that causes a rapid increase in intraocular pressure (IOP) and compression of the optic nerve, resulting in permanent visual field loss and optic nerve atrophy. APACG has rapid onset and apparent symptoms. The clinical manifestations include rapid loss of vision, nausea, vomiting, eye swelling, eye pain, ipsilateral migraine, and other symptoms, and in severe cases, blindness.^[1]^ The IOP of most patients can be controlled at the normal range after conventional clinical medication. However, in some patients, IOP is still poorly controlled after local and systemic IOP-lowering medications, and they still show signs such as persistently high IOP, congestion of the bulbar conjunctiva, corneal edema, shallow anterior chamber, and fixed mydriasis. Prolonged high IOP can cause permanent visual impairment and even retinal artery embolism in severe cases, affecting prognosis and increasing the incidence of postoperative complications.^[2,3]^ Therefore, in APACG patients with persistently high IOP that is not controlled by medication, the surgeon needs to perform surgical treatment as soon as possible to lower the IOP. This study retrospectively analyzed the treatment of patients with medically uncontrolled APACG. It also analyzed and compared the postoperative IOP, postoperative visual acuity, and incidence of complication rates between quadruple surgery and dual surgery, which is reported below.

## 2. Materials and Methods

### 2.1. Ethics

This retrospective study was conducted in accordance with the tenets of the Declaration of Helsinki. Informed consent for this study was waived due to the retrospective nature of this study. Informed consent for the operation was obtained from all patients.

### 2.2. Patients

#### 2.2.1. Selection and description of participants.

A retrospective analysis of 44 patients (45 eyes) who were treated in the Department of Ophthalmology, Shiyan Taihe Hospital between January 2019 and June 2021 was conducted. Patients were divided into 2 groups according to the surgical protocol. Group A comprised the quadruple procedure: posterior approach anterior segment pars plana vitrectomy + posterior capsulorhexis + phacoemulsification + intraocular lens (IOL) implantation + trabeculectomy. There were 20 patients, of which 4 were males and 16 were females. The patients were aged 48 years to 81 years and the mean age was 66.40 ± 7.27 years. Group B comprised dual surgery: phacoemulsification + IOL implantation + trabeculectomy. There were 25 patients, of which 8 were males and 17 were females. The patients were aged 51 years to 78 years and had a mean age of 67.12 ± 7.97 years.

The inclusion criteria were as follows: Patients diagnosed with APACG by specialist examination; Patients treated with local and systemic IOP-lowering drugs 72 hour preoperatively, with IOP ≥ 40 mm Hg or exhibiting high IOP, marked bulbar conjunctival congestion, corneal edema, shallow anterior chamber, and fixed mydriasis, and clinically determined to be unresponsive to drug treatment; Patients aged 40 to 81 years and without serious systemic diseases; Patients with complete medical records.

The exclusion criteria were as follows: Patients with severe organ disease and hepatic and renal insufficiency; Patients with incomplete clinical data; Patients with other types of glaucoma; Patients with comorbid fundus lesions or other ocular diseases (such as keratopathy); The eye has been previously treated with other surgical procedures.

### 2.3. Methods

#### 2.3.1. Preoperative examination.

All enrolled patients completed the following preoperative examinations: Systemic examination: routine blood and urine tests, coagulation function, chest fluoroscopy, electrocardiogram, Hepatic function and renal function tests. Specialist examinations: best corrected visual acuity (BCVA), slit lamp examination, IOP examination, visual field examination, A/B mode ultrasound examination, optical coherence tomography (OCT), fundus photography, anterior segment OCT, anterior chamber angle gonioscopy.

#### 2.3.2. Treatment.

After admission, all patients were administered IOP-lowering treatment: Systemic IOP-lowering drugs: 20% mannitol injection was administered intravenously based on 0.25 g to 2 g/kg of body weight. Topical IOP-lowering drugs: Pilocarpine eye drops, beta-blockers: timolol eye drops or carteolol eye drops, carbonic anhydrase inhibitors: brinzolamide eye drops, prostaglandin receptor agonists: latanoprost eye drops.

Surgery was performed after drug treatment failed and patients were divided into group A and group B. All operations are performed by an experienced surgeon.

1. Group A: quadruple surgery comprising posterior approach anterior segment pars plana vitrectomy + posterior capsulorhexis + phacoemulsification + IOL implantation + trabeculectomy; after the patient was routinely disinfected and draped for intraocular surgery, retrobulbar anesthesia was administered. A scleral puncture was made 4 mm behind the corneal limbus at the 10 o’clock position, and a 23G vitreous cutter was inserted in the absence of perfusion state to remove part of the anterior vitreous humor and then the anterior chamber was deepened. IOP, T-1 was measured by fingers. The bulbar conjunctiva was circumferentially cut at the angular scleral margin from the 12 o’clock to the 1 o’clock position, cauterized to stop bleeding, and a scleral flap with the angular scleral margin as the base was made. A 3.0-mm puncture knife was used to puncture the anterior chamber at the corneal limbus at the 11 o’clock position, and actovegin was injected. Circumferential capsulorhexis was performed, and a lateral incision was made at the 2 o’clock position. Phacoemulsification was performed after hydrodelineation and hydrodissection. The lens cortex was aspirated and the IOL was implanted. A portion of the trabecular tissue was excised and a perirhinal incision was made and then restored the iris. Scleral flaps were sutured intermittently. The aqueous humor flow was observed and seen to be flowing out at a visible and steady rate. Intermittent sutures were used to close the conjunctival wound. Posterior capsulorhexis was then performed through the scleral puncture opening with a vitreous cutter and the scleral puncture opening was sutured. At the end of the procedure, the eye was coated with tobramycin, dexamethasone eye drops (TobraDex S.A., Alcon Couvreur N.V., Belgium), ophthalmic ointment and bandaged.
2. Group B: dual surgery comprising phacoemulsification + IOL implantation + trabeculectomy: conventional intraocular surgery with disinfection and draping. Retrobulbar anesthesia was administered. The bulbar conjunctiva was circumferentially cut at the angular scleral margin from the 12 o’clock to the 1 o’clock position, cauterized to stop bleeding, and a scleral flap with the angular scleral margin as the base was made. A 3.0-mm puncture knife was used to puncture the anterior chamber at the corneal limbus at the 11 o’clock position, and actovegin was injected. Circumferential capsulorhexis was performed, and a lateral incision was made at the 2 o’clock position. Phacoemulsification was performed after hydrodelineation and hydrodissection. The lens cortex was aspirated and the IOL was implanted. Part of the trabecular tissue was resected, and a periradicular incision was made to restore the iris. Scleral flaps were sutured intermittently. The aqueous humor flow was observed and seen to be flowing out at a visible and steady rate. Intermittent sutures were used to close the conjunctival wound. At the end of the procedure, the eye was coated with TobraDex ophthalmic ointment and bandaged.
3. After surgery, patients in both groups were routinely treated with antibiotics and corticosteroid eye drops 4 times daily and maintained for 4 weeks to 6 weeks.

#### 2.3.3. Observation markers.

BCVA of the patient was measured preoperatively and 1 week, 1 month, 3 months, and 6 months postoperatively. BCVA was converted from the standard visual acuity table values to logMAR. The counting fingers, and hand motion were converted to 2.0 and 2.3, respectively.^[4]^ IOP measurement: preoperatively and 1 week, 1 month, 3 months, and 6 months postoperatively by using a Nidek NT-2000 non-contact IOP meter (Japan). The patient was placed in a sitting position, the head height was adjusted to eliminate neck compression, the head was placed in the anterolateral position, and each eye was measured 3 times. If the difference between the 3 measurements did not exceed 2 mm Hg (1 mm Hg = 0.133 kPa), then the average value of the 3 measurements was taken to calculate the IOP. Anterior chamber depth (ACD) measurement: preoperative measurement was made by the same technologist using a Tomey OCT scanner SS-1000 (Japan). The patient was placed in a sitting position, and the cornea was exposed as much as possible. The vertical distance between the center of the cornea to the crystal surface is the central ACD. Axial length (AL) measurement: Preoperative measurement was performed. The ultrasound probe frequency was adjusted to 10 mHz, each eye was automatically measured 10 times, and the average value was taken to calculate AL.

#### 2.3.4. Statistical analyses.

The SPSS 26.0 statistical software was used for data processing, and the quantitative data was described as mean ± standard deviation. Multiple linear regression analysis was used to analyze the influence of preoperative data (IOP, BCVA, age, gender, eyes, AL and ACD) on postoperative data. The 2-sample *t* test was used to compare the means between the 2 groups and the paired *t* test was used to the means before and after treatment in the same group. Qualitative data were expressed as (%) and the *χ*^2^ test was used for comparison. The test level was α = 0.05, and a difference of *P* < .05 was considered to be statistically significant.

## 3. Results

### 3.1. Multiple linear regression analysis for IOP and BCVA postoperatively

Multiple linear regression analysis showed that preoperative IOP and BCVA, age, gender, eyes, AL and ACD had no statistically significant influence on postoperative IOP (Table 1). There were statistically significant influence in preoperative BCVA on postoperative BCVA at 1 month, 3 months, and 6 months (*P*_1month_ = 0.007, *P*_3months_ = 0.007, *P*_6months_ = 0.007), but there were no statistically significant in other 6 independent variables (Table 2).

**Table 1:** Multiple linear regression analysis for intraocular pressure conditions postoperatively.

Factors	1 wk			1mo			3 mo			6 mo		
	β	t	*P* value	β	t	*P* value	β	t	*P* value	β	t	*P* value
BCVA	-0.344	-0.382	.70	-0.928	-1.562	.13	-0.326	-0.497	.62	-0.378	-0.545	.59
IOP (mm Hg)	0.024	0.589	.56	-0.038	-1.439	.29	-0.036	-1.218	.23	-0.059	-1.893	.07
Age (yrs)	-0.016	-0.197	.85	0.015	0.290	.77	-0.037	-0.637	.53	0.054	0.864	.39
Gender	-0.197	-0.153	.88	-0.913	-1.074	.29	-0.796	-0.849	.40	-0.815	-0.821	.42
Eyes	0.099	0.088	.93	-1.269	-1.701	.10	-1.635	-1.987	.05	-0.883	-1.014	.32
AL (mm)	1.119	0.985	.33	-0.098	-0.131	.90	0.609	0.737	.47	-0.020	-0.023	.98
ACD (mm)	-4.812	-1.837	.07	-2.349	-1.358	.18	-0.341	-0.179	.86	-1.626	-0.806	.43

ACD = anterior chamber depth, AL = axial length, BCVA = best corrected visual acuity, IOP = intraocular pressure.

Intraocular pressure and best corrected visual acuity were measured preoperatively. BCVA was converted from the standard visual acuity table values to logMAR.

**Table 2:** Multiple linear regression analysis for best correction visual acuity postoperatively.

Factors	1 wk			1 mo			3 mo			6 mo		
	β	t	*P* value	β	t	*P* value	β	t	*P* value	*β*	t	*P* value
BCVA	0.185	1.434	.16	0.301	2.833	.007	0.299	2.875	.007	0.284	2.837	.007
IOP (mm Hg)	-0.001	-0.124	.90	-0.010	-2.158	.04	-0.009	-2.014	.051	-0.009	-1.997	.05
Age (yrs)	0.013	1.136	.26	0.014	1.430	.16	0.010	1.076	.289	0.007	0.731	.47
Gender	0.291	1.576	.12	0.033	0.215	.83	0.012	0.082	.935	-0.022	-0.155	.88
Eyes	-0.263	-1.623	.11	-0.074	-0.550	.59	-0.101	-0.771	.446	-0.108	-0.861	.40
AL (mm)	0.039	0.239	.81	-0.090	-0.670	.51	-0.018	-0.141	.889	0.019	0.148	.88
ACD (mm)	0.227	0.605	.55	0.019	0.062	.95	0.135	0.446	.658	0.117	0.402	.69

ACD = anterior chamber depth, AL = axial length, BCVA = best corrected visual acuity, IOP = intraocular pressure.

Intraocular pressure and best corrected visual acuity were measured preoperatively. BCVA was converted from the standard visual acuity table values to logMAR.

### 3.2. Comparison of the general preoperative status

The general ocular status (age, eyes, gender, ACD, AL, BCVA, and IOP) of patients in group A were compared with that of those in group B preoperatively. The results showed that there were no statistically significant differences (*P*_age_ = 0.76, *P*_eyes_ = 0.20, *P*_gender_ = 0.37, *P*_AL_ = 0.94, *P*_ACD_ = 0.08, *P*_BCVA_ = 0.12) in age, eyes, AL, ACD, and BCVA before treatment between the 2 groups. The results showed that the mean IOP preoperatively was significantly higher in group A than in group B, and the difference between the 2 groups was statistically significant (*P* < .001) (Table 3).

**Table 3:** Comparison of the preoperative status.

	Group A	Group B	*P* value
Sample number	20	25	
Gender#			.37
Male	4 (20.0%)	8 (32.0%)	
Female	16 (80.0%)	17 (68.0%)	
Eyes#			.20
Right eye	9 (45.0%)	16 (64.0%)	
Left eye	11 (55.0%)	9 (36.0%)	
Age (yrs)*	66.40 ± 7.27	67.12 ± 8.00	.76
AL (mm)*	21.95 ± 0.37	21.94 ± 0.63	.94
ACD (mm)*	1.66 ± 0.23	1.79 ± 0.23	.08
BCVA*	1.46 ± 0.70	1.12 ± 0.71	.12
IOP (mm Hg)*	45.55 ± 11.41	27.32 ± 11.60	<.001

BCVA was converted from the standard visual acuity table values to logMAR.

# means chi-square test calculated *P* value,

* means 2-sample *t* test calculated *P* value.

ACD = anterior chamber depth, AL = axial length, BCVA = best corrected visual acuity, IOP = intraocular pressure.

### 3.3. Comparison of IOP postoperatively between the 2 groups

The IOP before and after treatment between the 2 groups was compared. The difference in the mean IOP between the 2 groups 1 week postoperatively was not statistically significant (*P* = .64). The mean IOP in group A was significantly lower than that in group B 1 month, 3 months, and 6 months postoperatively, and the difference was statistically significant (*P*_1month_ = 0.002, *P*_3months_ < 0.001,*P*_6months_ < 0.001) (Table 4).

**Table 4:** Comparison of intraocular pressure postoperatively between the 2 groups.

IOP (mm Hg)				
	1 wk	1 mo	3 mo	6 mo
Group A	14.55 ± 3.85	12.40 ± 1.64	12.55 ± 1.23	13.60 ± 1.10
Group B	14.08 ± 2.87	14.48 ± 2.33	15.24 ± 2.49	16.68 ± 2.58
t	0.470	-3.379	-4.727	-5.397
*P* value	.64	.002	<.001	<.001

The 2-sample *t* test calculated *P* value. IOP = intraocular pressure.

### 3.4. Comparison of BCVA postoperatively between the 2 groups

BCVA was compared 1 week, 1 month, 3 months, and 6 months postoperatively between the 2 groups. There was no statistically significant difference in BCVA between the 2 groups 1 week postoperatively (*P* = .66). The BCVA in group A was significantly higher than that in group B 1 month, 3 months, and 6 months postoperatively, and the difference was statistically significant (*P*_1month_ = 0.03, *P*_3months_ = 0.02, *P*_6months_ = 0.02) (Table 5).

**Table 5:** Comparison of best corrected visual acuity postoperatively between the 2 groups.

BCVA				
	1 wk	1 mo	3 mo	6 mo
Group A	0.90 ± 0.52	0.43 ± 0.17	0.31 ± 0.14	0.25 ± 0.14
Group B	0.83 ± 0.54	0.69 ± 0.54	0.576 ± 0.5294	0.50 ± 0.4992
t	0.450	-2.272	-2.413	-2.384
*P* value	.66	.03	.02	.02

The 2 sample *t* test calculated *P* value. BCVA was converted from the standard visual acuity table values to logMAR.

BCVA = best corrected visual acuity.

### 3.5. Comparison of IOP and BCVA preoperatively and 6 months postoperatively

Comparison of BCVA and IOP between the 2 groups preoperatively and 6 months postoperatively showed that the BCVA of both groups improved significantly at 6 months postoperatively compared with that preoperatively, and the difference was statistically significant (*P*_A_ < 0.001, *P*_B_ < 0.001). The IOP was significantly lower at 6 months postoperatively compared with that preoperatively, and the difference was statistically significant (*P*_A_ < 0.001, *P*_B_ < 0.001) (Table 6).

**Table 6:** Comparison of intraocular pressure and best corrected visual acuity preoperatively and 6 months postoperatively.

	IOP (mm Hg)		BCVA	
	Group A	Group B	Group A	Group B
Preoperatively	45.55 ± 11.41	27.32 ± 11.60	1.46 ± 0.70	1.12 ± 0.71
Postoperatively	13.60 ± 1.10	16.68 ± 2.58	0.25 ± 0.14	0.50 ± 0.4992
t	12.611	4.715	8.279	5.579
*P* value	<.001	<.001	<.001	<.001

The paired *t* test calculated *P* value. Postoperative follow-up time was 6 months after surgery. BCVA was converted from the standard visual acuity table values to logMAR.

BCVA = best corrected visual acuity, IOP = intraocular pressure.

### 3.6. Comparison of postoperative complications between the 2 groups

There were 4 postoperative complications in group A, of which 2 were cases of corneal edema, which gradually recovered after postoperative treatment; 1 case of shallow anterior chamber, which gradually deepened after active mydriasis; and 1 case of local choroidal detachment, which was cured after a period of oral hormone treatment and choroidal detachment at the postoperative review in 1 month. There were 14 postoperative complications in group B, including 2 cases of hypertension, 3 cases of inflammatory reaction, 6 cases of simple corneal edema, 2 cases of simple shallow anterior chamber, as well as 1 case of shallow anterior chamber and low IOP combined with corneal edema. The above patients’ symptoms improved after corresponding symptomatic treatments such as administering IOP-lowering and anti-inflammatory agents as well as administering agents to treat active mydriasis. A special case is a patient with corneal edema who developed postoperative eye grinding pain due to thin conjunctiva at the site of glaucoma filtration surgery, which did not improve with medication. In the end, the inferior conjunctiva of postoperative eye was taken for the conjunctival graft cover, and the symptoms improved. The incidence of surgical complications was 20% (4/20) in group A and 56% (14/25) in group B. The incidence of complications in group A was significantly lower than that in group B, and this difference was statistically significant (χ² = 6.000, *P* < .01).

## 4. Discussion

The prevalence of APACG in the Asian population is 10.4/100,000 to 12.2/100,000 per year, which is 2 to 5 times higher than that in the European and American populations,^[5]^ and the prevalence is highest in East Asia.^[6]^ APACG episodes were mostly seen in women,^[7]^ which is similar to the findings of the present study. Continuous high IOP leads to increased retinal and optic nerve susceptibility, and the pressure difference between the 2 sides of the lamina cribrosa directly compresses the optic nerve fibers and affects the microcirculation of the optic disc. The longer the high IOP lasts, the greater the pressure at the lamina cribrosa and the more severe the optic nerve damage and loss of visual function.^[8]^ Consequently, acute anterior optic nerve ischemia or central retinal vein obstruction can occur. This ultimately leads to irreversible visual impairment and increases the risk of surgical complications, including anterior chamber hemorrhage, vitreous detachment, ciliary block glaucoma, and suprachoroidal hemorrhage.^[2,3,9,10]^ Patients with APACG who are not treated with medication and have persistently high IOP need to undergo surgery as soon as possible to control the IOP. Anterior chamber puncture temporarily lowers IOP, relieves ocular ischemic symptoms, and facilitates restoration of ocular tissue circulation and perfusion pressure.^[11]^ However, in high IOP, the shallow anterior chamber and corneal edema will increase the difficulty of anterior chamber puncture. It is very easy to damage the cornea, iris and lens, and other structures; hence, releasing aqueous humor to lower IOP will increase the pressure difference between the anterior and posterior chambers simultaneously, which will, in turn, shift the position of the iris lens. This further aggravates the degree of the shallow anterior chamber, eliciting pathological changes and worsening the condition.^[12]^ Currently, ophthalmologists recognize the role of the lens in APACG, and phacoemulsification facilitates deepening of the anterior chamber and opening of the anterior chamber angle. However, APACG patients are prone to an increased surgical risk of iris prolapse, capsular membrane dilation, suspensory ligament rupture, posterior capsular membrane rupture, secondary vitreous detachment, and suprachoroidal hemorrhage due to high IOP. The surgical instruments are too close to the cornea, which aggravates damage to the corneal endothelial cells by ultrasound.^[13,14]^ Therefore, a combination of other surgical procedures is needed to address high IOP during phacoemulsification. Kong et al^[15]^ investigated intraoperative IOP-lowering drugs in patients with medically uncontrolled APACG and showed that aspiration of part of the vitreous humor lowered IOP with a lower probability of postoperative shallow anterior chamber, malignant glaucoma, and postoperative anterior chamber hemorrhage and inflammatory response than anterior chamber puncture. Recently, scholars study the pars plana vitrectomy in glaucoma surgery, and, in particular, the role of medically uncontrolled APACG, it can deepen ACD, reduce the difficulty of operation, decrease the incidence of the complications, reduce corneal endothelial injury.^[16]^

In addition, vitrectomy is less likely to result in complications such as vitreous hemorrhage and retinal detachment compared to vitreous aspiration.^[17]^ And, it can reduce the effect of ultrasound and anterior aqueous humor oscillation on the corneal endothelium, and obtain faster and better visual improvement.^[18]^ In group A, patients with glaucoma that did not respond to medical treatment had persistent high IOP, short ocular axis, shallow ACD. There is a high probability of the occurrence of postoperative malignant glaucoma. In patients with medically uncontrolled APACG, partial vitrectomy can lower IOP, deepen the anterior chamber, reduce ciliary ring block, and improve the success rate of surgery. In the present study, an intraoperative posterior approach to vitrectomy of the anterior segment was mainly used to reduce IOP. The conclusion shows that quadruple surgery is safer and more feasible compared with dual surgery as the former achieved lower IOP levels, better visual acuity, and lower incidence of surgical complications.

The main reasons can be attributed to the following points: 23G vitreous cutter was inserted in the absence of perfusion state to remove part of the anterior vitreous humor. The surgeon can control the steadily decreased IOP by judging the degrees of corneal edema and the hardness of the eye ball. This facilitates decreases the surgery risk due to a drastic decrease in IOP; The incidence of postoperative corneal edema was compared between the 2 groups: 20 cases in group A, 2 cases of corneal edema in group A, 7 cases of corneal edema in group B. The incidence of corneal edema was significantly lower in group A than in group B. The reasons are considered as follows: Vitrectomy is used to decrease posterior chamber pressure and balance the pressure difference between the anterior and posterior chambers, causing the protruding iris to flatten, deepening the ACD and decreasing operation space limitations for instrument manipulation and ultrasound energy^[16]^; It provides operating space for phacoemulsification devices to reduce corneal endothelial cell damage caused by mechanical damage, thermal burn from phacoemulsification energy, shock injury, and physical and chemical damage caused by Ringer’s fluid flushing during perfusion. Similarly, the multiple cases of corneal edema in the group B were related to these injuries caused by direct phacoemulsification in the operating space of the shallow anterior chamber. The anterior vitrectomy disrupts the integrity of the anterior vitreous membrane and, together with the posterior capsulorhexis, phacoemulsification and trabeculectom, deepen the anterior chamber, break the iridolenticular, ciliolenticular, iridovitreal and ciliovitreal blocks at the same time. In this study, with the establishment of external drainage channels for trabeculectomy, posterior capsulorhexis and the formation of peripheral iris holes, the group A could further block the aqueous humor block formed by the iridoposterior capsular, and reconstruct the pathway for the forward flow of atrial fluid in the vitreous cavity to avoid the occurrence of ciliary ring block glaucoma, which was suggested in the literature as early as 2012.^[19]^ In this study, the surgeon was able to control the rate and volume of vitreous incision according to the improved corneal transparency, the widened depth of the anterior chamber and the reduced IOP measured by the finger during the operation. Moreover, the surgical method was single-channel incision without perfusion, which had low interference degree to the vitreous cavity. It can largely avoid the occurrence of complications such as retinal detachment and vitreous hemorrhage caused by vitreous aspiration.^[16]^ There is none of them occurred in the present study. Comparing the occurrence of complications between the 2 groups established that the postoperative anterior chamber reaction was milder in the quadruple surgery group, and the incidence of anterior segment reactions such as postoperative corneal edema and inflammatory changes in the anterior chamber was lower. During vitrectomy, the inflammatory factors produced during the course of persisted high IOP can be drained through the vitreous cutter. Also, after lowering IOP, the degree of intraocular ciliary body congestion and edema was slightly alleviated, and the subsequent establishment of external trabecular channels and energy shock of phacoemulsification could promote the efflux of inflammatory factors. Theses may be the reasons for the mild postoperative anterior chamber inflammation in the group A.^[8]^ The majority of patients with medically uncontrolled APACG have completely closed anterior chamber angles, extremely shallow anterior chamber, and short axis length, which is consistent with the findings of the present study. Patients who still exhibit shallow anterior chamber and high IOP after anti-glaucoma surgery exhibit a clinical presentation that is consistent with malignant glaucoma.^[8,20]^ Therefore, compared to dual surgery, combined anterior vitrectomy deepens the preoperative ACD, avoids the occurrence of shallow anterior chamber, and reduces the probability of malignant glaucoma. Anterior vitrectomy can reportedly disrupt the integrity of the anterior vitreous membrane and reduce the resistance of vitreous humor flow to the posterior chamber.^[8,14,21,22]^ Further, the combined anterior and posterior segment surgery can effectively prevent the occurrence of malignant glaucoma by relieving the anatomical factors of ciliary ring block and anterior displacement of the iris septum of the lens and establishing a smooth channel for aqueous humor flow. Multiple linear regression analysis showed that preoperative BCVA was the influencing factor of postoperative BCVA at 1 month, 3 months, and 6 months. Considering that some patients may have unstable eye condition and poor visual acuity at 1 week postoperatively, it is possible that preoperative BCVA has no significant effect on postoperative BCVA at 1 week. Although the preoperative BCVA of group A was not significantly different from that of group B, the means of group A was worse than group B. However, the postoperative BCVA of group A was significantly improved compared with group B, which may be related to the effect of quadruple surgery on refractive status. The changes of refractive status were related to the influence of surgical incision on corneal curvature, the changes of anterior chamber, and refractive index on the accuracy of IOL refractive prediction, the size of capsulorhexis and the influence of posterior capsulorhexis on the stability of IOL.^[23]^ Partial anterior par plana vitrectomy can deepen ACD, and provided sufficient operating space for subsequent phacoemulsification. Sufficient operating space helps the surgeon to better control the size of the capsulorhexis to appropriate, and control the phacoemulsification energy and shorten the phacoemulsification time, and provides more conducive to the surgeon to adjust the position of the IOL. The above are conjecture, still need further research to confirm. However, this study is a retrospective study and lacks relevant data collection. Further exploration studies can be carried out in the future.

Some limitations that should be considered in this study are as follows: As our findings were not validated in a great amount of population, it is not known whether similar results arise when applied to larger populations. Because it was a retrospective study, some of the examinations failed to be taken, and some data will be further observed in the subsequent relevant studies, such as postoperative ACD, ocular axis length changes, and long-term changes of the optic nerve before and after surgery.

In the summary, compared with dual surgery, posterior approach anterior vitrectomy combined with posterior capsulorhexis, phacoemulsification, and trabeculectomy can achieve lower IOP levels, higher BCVA, and safer surgical outcomes. However, the effectiveness of quadruple surgery in preventing postoperative complications of malignant glaucoma in patients with high IOP and shallow anterior chamber in APACG still needs to be supported by data from numerous clinical studies. Consequently, it is still necessary to continue to observe the patients who undergo quadruple surgery at follow-up to understand the long-term results of this type of procedure.

## Author contributions

**Conceptualization:** Jie Qu, Yong Zhang.

**Data curation:** Jie Qu, Xin Li.

**Formal analysis:** Jie Qu, Zhen Tian.

**Funding acquisition:** Zhen Tian, Yong Zhang.

**Methodology:** Jie Qu, Xin Li, Yong Zhang.

**Writing – original draft:** Jie Qu.

**Writing – review & editing:** Zhen Tian.
